# Improvement of care for ICU patients with delirium by early screening and treatment: study protocol of iDECePTIvE study

**DOI:** 10.1186/s13012-014-0143-7

**Published:** 2014-10-02

**Authors:** Erwin Ista, Zoran Trogrlic, Jan Bakker, Robert Jan Osse, Theo van Achterberg, Mathieu van der Jagt

**Affiliations:** Department of Pediatric Surgery, Intensive Care Unit, Erasmus MC—Sophia Children's Hospital: University Medical Center Rotterdam, Rotterdam, 3000 CB The Netherlands; Department of Intensive Care Unit, Erasmus MC: University Medical Center Rotterdam, Rotterdam, The Netherlands; Department of Psychiatry, Erasmus MC: University Medical Center Rotterdam, Rotterdam, The Netherlands; Radboud University Medical Center, Scientific Institute for Quality of Healthcare, Nijmegen, The Netherlands; Center for Health Services and Nursing Research, KU Leuven, Leuven Belgium

**Keywords:** Intensive care, Critical care, Delirium, Screening, Delirium management, Implementation, Guideline

## Abstract

**Background:**

Delirium in critically ill patients has a strong adverse impact on prognosis. In spite of its recognized importance, however, delirium screening and treatment procedures are often not in accordance with current guidelines. This implementation study is designed to assess barriers and facilitators for guideline adherence and next to develop a multifaceted tailored implementation strategy. Effects of this strategy on guideline adherence as well as important clinical outcomes will be described.

**Methods:**

Current practices and guideline deviations will be assessed in a prospective baseline measurement. Barriers and facilitators will be identified from a survey among intensive care health care professionals (intensivists and nurses) and focus group interviews with selected health care professionals (*n*?=?60). Findings will serve as a foundation for a tailored guideline implementation strategy. Adherence to the guideline and effects of the implementation strategies on relevant clinical outcomes will be piloted in a before-after study in six intensive care units (ICUs) in the southwest Netherlands. The primary outcomes are adherence to screening and treatment in line with the Dutch ICU delirium guideline. Secondary outcomes are process measures (e.g. attendance to training and knowledge) and clinical outcomes (e.g. incidence of delirium, hospital-mortality changes, and length of stay). Primary and secondary outcome data will be collected at four time points including at least 924 patients. Furthermore, a process evaluation will be done, including an economical evaluation.

**Discussion:**

Little is known on effective implementation of delirium management in the critically ill. The proposed multifaceted implementation strategy is expected to improve process measures such as screening adherence in line with the guideline and may improve clinical outcomes, such as mortality and length of stay. This ICU Delirium in Clinical Practice Implementation Evaluation study (iDECePTIvE-study) will generate important knowledge for ICU health care providers on how to improve their clinical practice to establish optimum care for delirious patients.

**Trials registration:**

Clinical Trials NCT01952899

**Electronic supplementary material:**

The online version of this article (doi:10.1186/s13012-014-0143-7) contains supplementary material, which is available to authorized users.

## Background

Delirium, also known as ‘brain failure’, is a common form of vital organ failure in critically ill patients. It has an acute onset and is characterized by a combination of attention and cognitive deficits and a fluctuating consciousness [1]. Disturbed motor activity (apathy or agitation), visual hallucinations, and sleep disruption are among the most frequently observed symptoms. The reported incidence of delirium in critically ill patients ranges from 16%-89%, depending on type of intensive care unit (ICU), method of assessment, and patient population [2]. Delirium is especially common in over 65-year-old patients [3],[4]. Delirium is an important, independent predictor of mortality [5]-[7]. Critically ill patients may develop delirium-associated complications leading to serious self-harm, such as attempting to remove the endotracheal tube, central lines and catheters, or falling out of bed [8]. Many delirious patients show severe psychological distress and anxiety [8]. Delirium is a cause of longer ICU and hospital stay, and affected patients have more long-term morbidity [2],[5] and a worse prognosis after discharge compared with non-delirious ICU patients. The duration of delirium is also an important prognostic indicator for various adverse outcomes. Furthermore, recent research suggests that ICU delirium independently predicts long-term cognitive impairment comparable to mild Alzheimer's disease [5],[7],[9]-[14]. The sequelae associated with delirium are a cause of increased health care costs [15].

Therefore, delirium in these critically ill patients requires adequate management, including systematic screening to prevent that the diagnosis is missed in patients who display only subtle signs of delirium (‘hypoactive delirium’) [16]. The importance of routine screening for delirium at the ICU was already advocated in the clinical practice guidelines for pain and sedation issued in 2002 by the American College of Critical Care Medicine (ACCM)/Society of Critical Care Medicine (SCCM) [17] but delirium screening has not yet been widely adopted [18].

The Netherlands Society for Intensive Care (NVIC) developed and authorized a delirium guideline in 2010 [19]. The recently published ‘Clinical Practice Guidelines for the Management of Pain, Agitation and Delirium (PAD) in the ICU’ from ACCM/SCCM [20] are generally in line with this guideline. Both guidelines recommend routine delirium screening in critically ill patients using a valid and reliable screening tool. Despite this, a validated delirium screening tool is not routinely used in most Dutch ICUs; the management of delirium strongly depends on local policy and is generally not in line with current recommendations [16],[21]. The Netherlands is not alone in this respect; also in other countries, the attention paid to the monitoring and management of ICU delirium has been shown to be insufficient [18].

‘Get With The Guidelines’ initiatives have the potential to accomplish practice changes in the ICU environment that may result in improved clinical outcomes, including mortality [22]. However, the most effective way to translate such ‘paper’ guidelines to real-life clinical practice is not clear. In general, a variety of barriers may be in the way of good adherence to guidelines and interventions [23]-[25]. Hence, it is necessary to develop a tailored implementation strategy based on a thorough analysis of the context and target group [24].

### Objective

We designed the ICU Delirium in Clinical Practice Implementation Evaluation (iDECePTIvE) study with the following aims: 1) to assess the barriers and facilitators for adherence to the Dutch ICU delirium guideline [19]; 2) based on these results, to develop a tailored implementation strategy targeting these influencing factors for successful implementation and long-term adherence to the guideline; and 3) to study the effects of tailored implementation on adherence to the guideline, clinical outcome, and costs in a prospective multi-center study.

The following research questions are addressed to answer these aims:

1. What are the current practices (before-implementation) with regard to delirium management and degrees of adherence to the delirium guideline in the participating ICUs?
2. What are the influencing factors (barriers and facilitators) for the implementation of the ICU delirium guideline in the ICUs as reported by intensivists, ICU nurses, and psychiatrists?
3. What should be the content of a tailored implementation strategy to improve adherence to the delirium guideline based on the answers to the first two questions?
4. What is the effect of the tailored implementation strategy on guideline adherence, knowledge of health care providers, delirium incidence, clinical outcomes (mortality, length of ICU stay) and health care costs?
5. What are potential explanations for why the intervention was effective or not, based on ICU and health care providers' characteristics indicative of local ‘culture?’

## Methods

**Figure 1:**
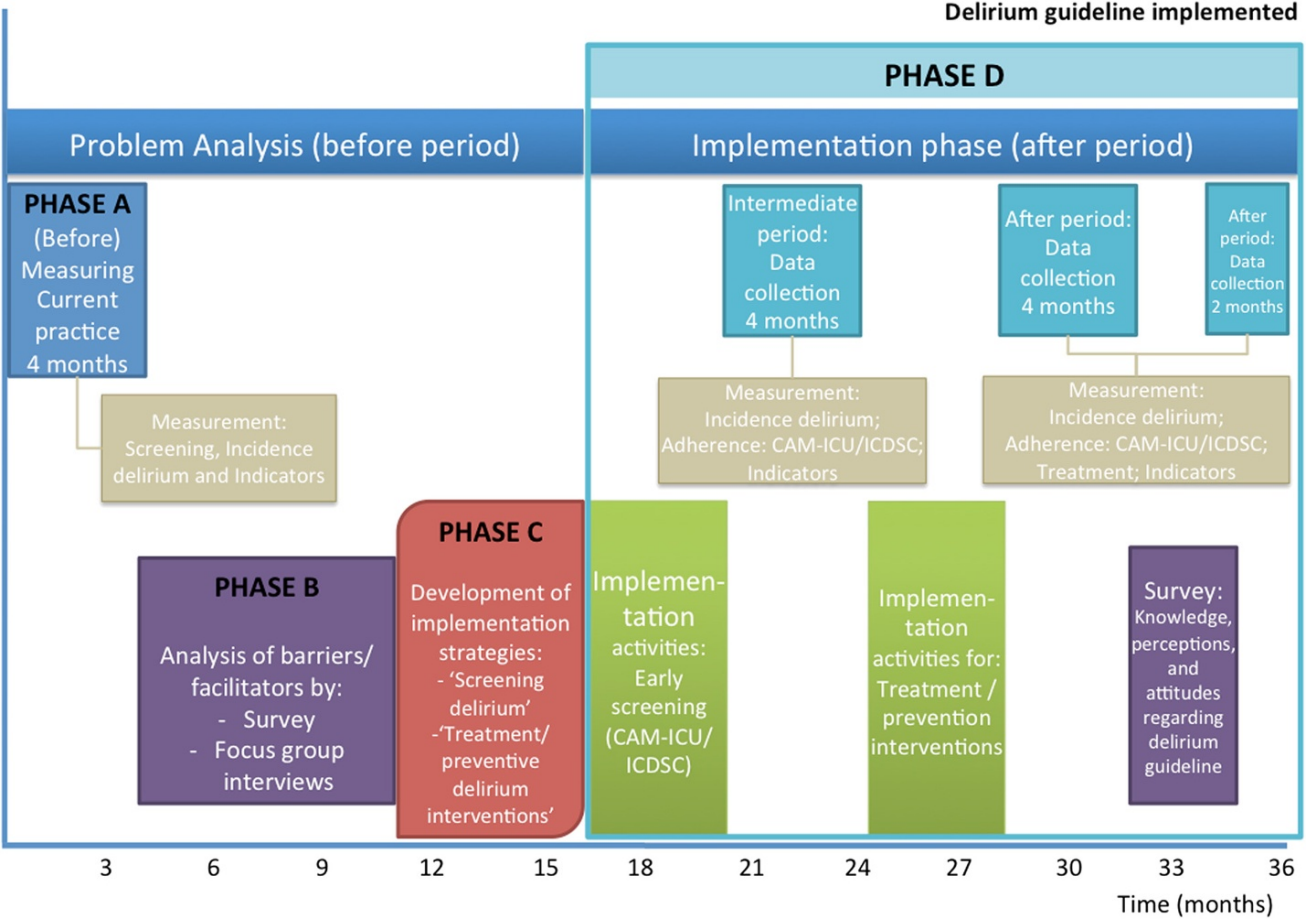
**Study schedule.**

The iDECePTIvE study is a descriptive, explorative prospective multi-center study, using a mixed method design in six ICUs in the southwest of the Netherlands. In line with the research questions, we designed the study in several phases (see detailed schedule in Figure 1):

A. Analysis of the current practice of delirium management and level of adherence to the Dutch NVIC delirium guideline in the participating ICUs.
B. Identification of barriers and facilitators for the implementation of the ICU delirium guideline.
C. Development of a tailored implementation strategy based on the results of phases A and B.
D. Implementation of the guideline and measurement of the effects.

We describe the methods, population, analysis, and outcomes per study phase. An overview is given in Table 1 and Figure 1.

**Table 1:** **Overview of study phases**

Phase	Research question	Methods	Target population/data resource	Measures
A	What are the current practices (before-implementation) and the adherence to the delirium guideline in the participating ICUs?	Prospective, descriptive study, analyzing variation of care	Data from 6 ICUs	Indicators e.g.:
				??-Adherence to delirium screening
				??-Incidence of delirium
				??-Pharmacological treatment
				??-Sedation practices
				??-Non-pharmacological treatment
				??-Knowledge
B	What are the influencing factors (barriers and facilitators) for the implementation of the Dutch ICU delirium guideline by intensivists, ICU nurses, and psychiatrists?	Survey on knowledge, attitudes and perceptions, and structured focus group interviews	Health care professionals: intensivists, residents, ICU nurses, managers and psychiatrists, geriatrist or neurologist	Barriers and facilitators classified as related to: 1) guideline; 2) provider characteristics (e.g. knowledge and attitudes); 3) institutional characteristics (e.g. organization, structure, resources); 4) implementation (e.g. how and to what extent the guideline is implemented); 5) patient characteristics; and 6) social context (e.g. ICU culture).
C	What is the content of a tailored strategy to improve the adherence to the delirium guideline?	Strategy development according to implementation frameworks by Grol and Wensing, and Cabana	Matching the data from the current practice, questionnaires and focus groups and questionnaires to construct effective implementation strategies from the literature	Tailored multifaceted implementation strategy to effectively implement current guideline based delirium management
D	What is the effect of the tailored implementation strategy on guideline adherence, knowledge of health care providers, delirium incidence, clinical outcomes (mortality, length of stay) and health care costs?	Prospective before-after study	Data from 6 ICUs	(Process) indicators e.g.:
				-Adherence delirium screening
				-Incidence of delirium
				-Pharmacological treatment
				-Non-pharmacological treatment
				-Knowledge
				Outcomes e.g.:
				-Length of stay
				-Hospital mortality
				Costs
D	Explore potential explanations for why the intervention was effective or not based on ICU and health care providers' characteristics indicative of local ‘culture’.	Process evaluation: qualitative (outcomes,) and quantitative data (survey and interviews)	Data from 6 ICUs. Frame work for process evaluation, matching outcomes with actual exposure, and experiences of the implementation strategy	Underlying mechanisms that explain the effects of the study.

### Study sites and participants

The study will be performed in six ICUs of university, non-university-teaching, and non-university-non-teaching hospitals. Wards were selected to include several levels of intensity of intensive care practice. Inclusion criteria for patients are: age ?18 years and admitted to an ICU for ?24 h. Involved professionals are all ICU physicians and nurses.

### Phase A: analysis of current practice of delirium management and adherence to the Dutch delirium guideline

#### Study design and population

Over a 4-month period, we will prospectively record the incidences of delirium, frequency of delirium assessments, types of pharmacological and non-pharmacological treatments, and documented preventive interventions. Unit staff will not be actively informed about the study, nor will they be educated on delirium, so as to avoid a Hawthorne effect as much as possible. The results of this analysis will serve as a baseline measure to compare future practice and outcome changes in the course of the implementation project.

#### Measures

Adherence to and deviation from the delirium guideline will be assessed using the following indicators. The primary outcome in this study phase is the percentage of patients screened with either the Confusion Assessment Method for the Intensive Care Unit (CAM-ICU) [26] or the Intensive Care Delirium Symptoms Checklist (ICDSC) [27], which both are validated for use in the ICU. Adherence is defined as screening of every eligible patient at least once per nursing shift (i.e. three times daily). The secondary outcomes are pharmacological treatment with haloperidol or other antipsychotic drugs; documented psycho-hygiene measures aimed at preventing delirium (such as use of hearing aids or glasses and stimulating a proper night-day rhythm; early mobilization and physiotherapy). Delirium is defined either as a positive CAM-ICU or ICDSC score, or if a screening tool is not used, pragmatically defined as 1) administration of haloperidol or other antipsychotic drug; or 2) delirium reported by a physician or ICU nurse in the patient record, as confirmed by a designated research nurse on site. Data on adherence to these indicators for all ICU patients will be collected by various methods: direct observations and systematic registration in the patient data management system, medical records, and 24-h ICU-care lists.

#### Analysis

Descriptive statistics will be used to describe the outcomes. Multivariate analysis serves to compare ICUs regarding patient mix (e.g. age, diagnosis, severity of illness [Acute Physiology and Chronic Health Evaluation, APACHE II score]) and ICU level of care. The incidence of delirium will be calculated based on screening (CAM-ICU or ICDSC) and medical notes (physicians and nurses) and consulting experts (psychiatrist, geriatrists, or neurologist).

### Phase B: identification of barriers and facilitators for the implementation of the ICU delirium guideline

#### Study design

Barriers and facilitators will be identified with quantitative and qualitative research methods: 1) a survey and 2) in-depth focus group interviews. The main aim is to understand, and where possible explain, the opinions, attitudes, beliefs, and perceived practices of health care professionals with regard to delirium in critically ill patients [28].

#### Survey

ICU physicians and ICU nurses will be surveyed on their beliefs, attitudes, and practices regarding the incidence, clinical relevance, screening for, treatment, and prevention of delirium. The survey will be partly based on the instrument developed by Ely et al. [29] and expanded with self-developed questions on non-pharmacological and preventive interventions for delirium. Furthermore, the questionnaire will contain statements about the delirium guideline and attitude towards guidelines in general [30] and questions assessing knowledge [29],[31],[32] and demographic characteristics of responders. The survey will be repeated in a later phase (D, after implementation) to assess impact of implementation on attitudes and practice perceptions.

#### Focus group interviews

The uniqueness of a focus group interview is its ability to generate data based on the synergy of group interaction. This type of analysis is also essential to understand the potential barriers and facilitators in the collaboration between health care professionals, e.g. nurses and physicians.

An interview framework and protocol will be developed with a series of open-ended questions, based on the framework of knowledge-attitude-behavior related barriers for guideline adherence of Cabana et al. [23]; the interdisciplinary conceptual framework of clinicians' compliance with guidelines of Gurses et al. [33]; and the framework for adherence to clinical practice guidelines in the ICU of Cahill et al. [34]. These frameworks distinguish six major categories of factors that influence adherence to evidence-based guidelines: 1) the guideline; 2) the health professionals' characteristics (e.g. knowledge and attitudes); 3) the institutional characteristics (e.g. organization, structure, resources); 4) the implementation (e.g. how the guideline is implemented); 5) the patient characteristics; and 6) the social context (e.g. ICU culture). The survey findings will be discussed in the focus group interviews to explore discrepancies between professionals' beliefs and daily practices.

#### Study population

All health professionals in the six ICUs, including ICU nurses, intensivists, residents, and psychiatrists or geriatrists, will be asked to complete an online survey.

For the focus group interviews, we will purposefully select 8-10 professionals involved in delirium care from each participating ICU, e.g. intensivists, residents, ICU nurses, managers and psychiatrists, geriatrist, or neurologist.

#### Outcome measures

Barriers and facilitators for adherence to the delirium guideline in daily practice will be classified according to the six major categories of the above-mentioned frameworks [23],[33],[34]. Combining the findings on current practices (phase A) with the results of the surveys and focus group interviews (phase B) will give a complete overview of current practices, attitudes, and perceptions at baseline of the study and potential barriers and facilitators for implementing the guideline.

#### Analysis

The different barriers and facilitators will be quantified and expressed in percentages. Continuous data will be presented as means (+/?SD), non-normally distributed as medians (interquartile range). Differences among the health care professionals and across the six ICUs will be evaluated with ANOVA or Kruskall—Wallis test depending on normality of data distributed. Data will be analyzed using IBM SPSS version 21.0.

The focus group interviews will be audiotaped and transcribed in full for analysis. Qualitative analysis will be done with the software package Atlas.ti using Krueger's framework analysis approach, which provides a clear series of steps: familiarization, identifying a thematic framework, indexing, charting, and mapping and interpretation [35]. To strengthen validity of the analysis, participants will be invited to provide feedback on a summary of the focus group interview.

### Phase C: development of the tailored implementation strategy

The implementation model of Grol et al. [24] assumes that the effectiveness of the implementation is enhanced if the chosen strategy is appropriate to the innovation, the setting and target group, and includes an assessment of current practice and of barriers and facilitators for guideline adherence [36]. In this study, we will use this model, which includes several steps. Step 1 involves the development and clear description of the recommended performance. Steps 2 and 3 analyze the setting and target group. Both current practice and the barriers and facilitators for guideline adherence are explored in these steps. Step 4 involves developing and choosing strategies and measures to change practice that target the previously identified barriers and facilitators. Steps 5 and 6 subsequently develop and apply the implementation to integrate changes in routine of care, and step 7 evaluates the implementation strategy [24].

Based on the results of phases A and B, a team of implementation experts, investigators, and clinicians (nurses and physicians) will develop a tailored strategy for implementation aimed at enhanced delirium guideline adherence, focusing on the barriers and facilitators most frequently encountered. The strategy should facilitate integration of the guideline in daily practice and its sustained use over time. The expert team will discuss the content of the tailored implementation strategy with local ICU teams. Two main questions should be answered in this setting: 1) Can the barriers and facilitators found be successfully translated into tailored implementation interventions?; and 2) Are the tailored interventions applicable in daily practice? Finally, the implementation expert team will adapt the tailored strategy based on feedback provided by the local ICU teams.

Tailored multifaceted strategies are likely to be more effective than single strategies [36]. Barriers and facilitators are expected to exist at different levels. This means that the tailored strategy will consist of a combination of different interventions targeted to influence the professionals, the organization, and the structure of care. To strengthen the strategy development, we will be building upon existing theories for behavioral change like social learning or social influencing theories [37],[38]. Finally, the selected implementation interventions will be matched to evidence-based interventions, described by the EPOC taxonomy [39]. We give some examples to illustrate our approach. Possible barriers at a professional level are aspects of hierarchy and lack of collaboration between nurses and doctors. A physician may have doubts and not start treatment after an ICU nurse has identified a delirious patient. This may discourage nurses to screen for delirium on a daily basis. A consistent management protocol could properly remove this barrier by linking screening results to a treatment. Another potential barrier is the perceived time-consuming nature of routine screening. ICT solutions to facilitate registration could be helpful in this regard.

### Phase D: implementation study

#### Study design and population

The impact of implementation of a delirium guideline in six ICUs for adults will be studied in a pilot feasibility study using a prospective multi-center before-after study design (Figure 1). The primary aim will be to evaluate to what extent a guideline implementation program can achieve changes in ICU professionals' clinical practice with regard to delirious patients. This will be measured by the degree of adherence to the guideline recommendations. A secondary aim will be to evaluate the impact of the implementation interventions on clinical outcomes (hospital mortality and length of stay at ICU) and costs of the implementation and whether these may be linked to the practice changes achieved. A before-after study is considered a useful instrument, particularly for pilot studies in which interventions are initially evaluated and refined if necessary before the testing of the implementation strategy on a wider scale is justified.

Implementation of the delirium guideline will be two-phased. First, we will implement delirium screening with the CAM-ICU or ICDSC. This is an essential first step because prevention and treatment of delirium will only be possible after adequate and early recognition. Second, protocolled prevention and treatment interventions (pharmacological and non-pharmacological) will be implemented. ICUs will be free to select either tool based on local preference.

#### Before period—intermediate period—after period

We have defined three periods (see Figure 1). The first is the four-month *before* period, during which delirium will be assessed as described earlier (phase A, current practice evaluation), i.e. on the basis of antipsychotic drug therapy and documented delirium diagnosis as a proxy for delirium incidence when no systematic screening is performed. The second period is the four-month *intermediate* period after implementation of delirium screening with the CAM-ICU or ICDSC. The same data as in the before period will be collected, and in addition delirium incidence as measured with the CAM-ICU or ICDSC. This period serves to assess the impact of the barrier analysis (phases A and B) and screening implementation *without* formal implementation of a prevention and management protocol*.* The third period is the *after* period, in which the process measures (adherence to screening, prevention, and pharmacological and non- pharmacological) and clinical outcomes will be studied in two successive four-month and one two-month period (see Figure 1).

#### Survey

Post implementation of the survey previously done in Phase B will be repeated to explore changes in knowledge, attitude, perceptions, current beliefs, and perceived practices regarding delirium management of intensivists, physicians, and ICU nurses from the participating ICUs [29],[31],[32].

#### Main outcome measures

The primary outcomes of the prospective before-after pilot implementation study are adherence to screening and (non)pharmacological treatment as described in the Dutch ICU delirium guideline. Adherence to the delirium screening procedure will be calculated as the percentage of performed assessments per day, relative to the total number of assessments that should have been performed (i.e. a minimum of three times daily in every patient). Successful implementation is defined as adherence to assessment of more than 85%. Delirium experts (expert raters) will conduct accuracy spot-checks during the intermediate and after periods on a random sample of the bedside nurses' screening assessments. The expert will then share his or her findings from the CAM-ICU or ICDSC assessment with the bedside nurse and point out any mistakes or misconceptions in the nurse's assessment. Cohen's kappa and 95% CIs will be used to analyze agreement of CAM-ICU/ICDSC assessments between the bedside nurses and the delirium experts.

Adherence to the following aspects of non-pharmacological and/or pharmacological interventions and prevention interventions (based on the guideline) will be assessed: a) pharmacological: prescription of antipsychotic drugs (e.g. haloperidol); b) non-pharmacological: attention to orientation, prevention of sleep deprivation, and the use of glasses and hearing aids; and c) prevention: adherence to early mobilization and physiotherapy. Data on adherence indicators will be collected from systematic registration in the patient data management system and direct observations.

The secondary outcomes are the process measures (as defined in the section process evaluation, e.g. incidence of delirium; delirium knowledge of nurses and physicians; interrater reliability of delirium assessment (CAM-ICU or ICDSC); hospital mortality in the before, intermediate, and after periods).

#### Other variables

During all measurement periods, data will be collected on: psychoactive drugs (psychiatrist, neurologist, or geriatrician consultations), complications (self-removal of endotracheal tube, central lines, feeding tubes, and falls out of bed) and length of ICU stay, length of hospital stay, mortality, and institutionalization after hospital discharge. These data are needed to explore a cost benefit analysis of completed implementation. Furthermore, severity of illness scores (APACHE II score) and ICU ward specialty (e.g. internal medicine, surgery, or combined) will be retrieved from the Dutch National Intensive Care Evaluation (NICE) registry with consent from the participating ICUs.

#### Analysis

Results are expressed as percentages. Adjusted analyses will be done using repeated measures analysis for binary outcome data. Finally, outcome differences between the ICUs adjustments for patient mix (e.g. age, diagnosis, APACHE II score) and ICU level will be assessed using multi-variable analysis.

#### Sample size

Based on the literature, the adherence rate to screening with the CAM-ICU or ICDSC could increase from 70%-85%, following implementation [31],[40]. Consequently, the sample size will be 924 patients (231 patients in the before period and 693 in the after period (3 periods, Figure 1)). The alpha level of significance is set at 0.01 (two-tailed) and the beta level at 0.90.

#### Process evaluation

A process evaluation can give insight into determinants or indicators of potential success or failure of a tailored implementation strategy [41],[42]. For this purpose, process data will be collected for each of substrategies within the ‘tailored strategy’.

We will conduct in-depth qualitative interviews with clinicians (*n*?=?12) from participating ICUs to understand their perceptions of the study’s effect on local practice and the effectiveness of individual components of the intervention. We will recruit these individuals by invitation letters sent to all six ICUs. A semi-structured interview guide will be developed to facilitate the interviews. The process evaluation will provide insight in elements of the tailored strategy that are less feasible and need refinement before further implementation. In a post-implementation survey, we will examine whether earlier barriers are removed and facilitators are taken up.

#### Process measures

- Education: number of nurses attending per ward, duration of training per ward, evaluations of nurses attending the training, experience with the training;
- Tailored strategy: elements of the strategy are delivered as agreed; feasibility of the strategy, user experiences with the strategy, degree to which barriers are solved, and facilitators are used.

Other process indicators will be defined after the strategy procedure has been developed.

Data will be collected from questionnaires, interviews, and direct observations. The process indicators will be related to relevant outcomes (e.g. mortality reduction) of the ‘tailored strategy’ to identify elements of the strategy that were particularly associated with the success of the implementation.

#### Economic evaluation

Prolonged admission on the ICU due to delirium is related with increased health care costs. Therefore, strategies that focus on increasing adherence with the Dutch delirium guideline are likely cost-effective [15]. The economic evaluation compares usual care (before) and care after implementation of the guideline. The aim of this analysis is to explore whether the likely overall cost saving from the tailored guideline implementation strategy exceeds the overall cost of the tailored guideline implementation process.

#### Cost analysis

The economic evaluation will be performed from a health care perspective and in accordance with guidelines for such analysis [43]. Care costs of each strategy are defined as all direct medical costs associated with procedures performed within that strategy. The resources consumed by the implementation strategies will be assessed in the clinical study by collecting data on personnel costs (time spending for the strategy delivery team, for the nurses attending the strategy related activities, and for systematic screening), material costs (antipsychotic drugs), and overhead costs. Medical costs will be estimated by multiplying resource utilization with the cost per unit of resource (market prices, guideline prices, or self-determined prices based on costing methods, i.e. full costing) [43]. The implementation process and consequent costs will be estimated by focusing on activities performed with costs accumulated at the activity level(s) of the health care implementation processes. The incremental costs will be determined by the difference in resource consumption between usual care and tailored implementation. The economic analysis will be a cost-minimalization analysis, in which we investigate whether the likely overall cost saving from the tailored guideline implementation strategy exceeds the overall cost of the tailored guideline implementation process.

### Ethical considerations

This study protocol was presented to the Medical Ethical Committee of the Erasmus University Medical Center (registration number: MEC-2012-063). An exemption was obtained as ethical approval for this type of study is not required under Dutch law. This study is registered in the Trial register, located at http://clinicaltrials.gov, under number: NCT01952899. Data collection will be in line with Dutch METC endorsed privacy regulations, ensuring that data collected for the analyses cannot be traced to individual patients by the coordinating investigators because the data will be anonymized by the local investigators who provide the data.

## Discussion

The goal of the iDECePTIvE study is to identify barriers and facilitators for adherence to a national ICU delirium guideline. We will analyze the current practice (Phase A) before executing the survey and focus group interviews to avoid a possible Hawthorne effect (attention effect) by which members of the focus groups could be influenced. Based on these results, a tailored implementation strategy targeting these influencing factors will be developed for successful implementation and long-term adherence to the guideline. Finally, a before-after multicenter study will be conducted to evaluate the impact of the implementation strategy on clinical practice including a cost-effectiveness analysis and the effects on clinical outcomes.

This study is unique in that it includes all components of a multifaceted implementation in a large cohort of critically ill patients and includes measurement of important clinical outcomes based on a national database benchmarking outcomes of intensive care in the Netherlands. In a systematic review of the literature, we found that ICU delirium implementation studies mainly focus on implementation of screening or assessment tools for early recognition of delirium in ICU patients and tend to ignore improvement of prevention and treatment [44]. Most implementation strategies were not based on a systematic analysis of the context, including barriers and facilitators. Studies have shown that large-scale implementation of a delirium screening tool in the ICU is both feasible and sustainable with a compliance rate that may exceed 80% [31],[40],[45]-[47]. However, these studies focused only on screening and not on pharmacological and non-pharmacological treatment of delirium. Furthermore, the analysis of the barriers and facilitators was unstructured and not focused on treatment as proposed in the current delirium guideline. In this proposed study, the multifaceted strategy will be based on theoretically underpinned mechanisms to accomplish improved adherence to a guideline on ICU delirium. A study including all these components and of this magnitude has not been performed previously. Also, outcome assessments and cost-effectiveness analysis have not been performed on this scale.

Furthermore, the results of this study will expectedly provide us with further knowledge on effective implementation of optimal care of the delirious patient at the ICU. We will provide answers to not only the ‘why should we implement’ questions, but also answers to ‘how to implement’ question and provide clues to reproducibility. In other words, the results of this study may help persuade clinicians and nurses to put effort into formal implementation of interventions, when indeed the results confirm that these may improve outcomes of our patients.

The results of this project will therefore add to the general body of knowledge about implementation science at the ICU. The knowledge generated from this study can also be of use in other improvement projects and guidelines in the ICU that require collaboration between different health care providers [48],[49].

A major limitation of this study with regard to the clinical outcomes assessment (mainly: mortality) is the before-after study design (phase D). Although changes in team behavior and clinical practices (i.e. guideline adherence; the primary outcome) related to delirium management during the course of this study are very likely to be due to the implementation itself, changes in mortality (secondary outcome) are less likely to be caused exclusively by the implementation. Other factors besides the implementation interventions that may impact on mortality include case-mix changes over the course of this study, changes in composition of the medical teams, or organizational changes (e.g. rebuilding of ICU). Such changes can only be partly accounted for in multivariable analysis because unmeasured (or unmeasurable) confounders are potential sources for bias. Therefore, results of the pilot before-after study on clinical outcomes rather than process measures should be interpreted with great caution. The generalizability is limited because concurrent changes in content or organization of care that may influence clinical outcomes may confound attribution of observed changes in outcomes to the implementation strategy. Furthermore, there is some evidence that suggests that uncontrolled before-after studies may overestimate the effects of quality improvement projects like this [24],[50]. In future studies, a stepped wedge cluster randomized trial would be a more sophisticated design, in which at the end of the study all participants will have received the intervention [51]. However, the current study with the proposed design will provide details regarding the feasibility of establishing practice changes and guideline adherence improvements with a tailored implementation and provide valuable information on successful and less successful implementation interventions and the need for their refinements in future studies on a wider scale. Future implementation studies aimed at improving outcomes will likely benefit from the knowledge generated by our study because effective interventions to change practice will be identified, which is a first essential step towards outcome improvement.

We hypothesize that the incidence rates of delirium in ICU patients will increase after implementation of early screening. One of the main reasons is that hypoactive delirium will be detected, which is ill-recognized without systematic screening. On the other hand, implementation of prevention and management of delirium is expected to decrease incidences. The balance between these opposite forces may explain why some studies found decreased incidences and others increased incidences of delirium after implementation of interventions targeted at delirium. Therefore, we propose a two-phased implementation process (Phase D: first screening implementation, thereafter prevention and treatment). After data collection for this reference period (before intermediate period), guideline-recommended treatment will be implemented. This approach prevents strong bias in the comparison of the incidence rates between the intermediate and after periods because assessment of delirium before and after implementation is similar.

The ultimate aim of our study is to reduce the incidence of delirium and improve the outcome for ICU patients and their families by implementing the national and international evidence-based guidelines on ICU delirium management. Furthermore, this study provides a framework for future efforts to stimulate guideline adherence and delirium management.

## Authors’ contributions

EI and MJ designed the study protocol and EI, MJ, and ZT wrote the manuscript. EI and MJ will coordinate the study as principal investigators and ZT will carry out this multicenter study. All authors are member of the study group. All authors read and approved the final manuscript.

## Authors’ information

ZT, MSc, nurse scientist, ICU nurse, *investigator-PhD student,* Erasmus MC

Professor JB (MD PhD), internist-intensivist, vice-chair, ICU Erasmus MC

MJ, MD, PhD, neurologist-intensivist, ICU Erasmus MC

EI, RN, PhD, implementation fellow, nurse scientist, Erasmus MC

RJO, MD, PhD, psychiatrist, Erasmus MC

Professor TA, RN PhD, Nurse scientist, IQ Healthcare UMC St Radboud, Nijmegen

MEC-number: MEC-2012-063

ZonMw grant number: 171203008

## Authors’ original submitted files for images

Below are the links to the authors’ original submitted files for images. Authors’ original file for figure 1

## Abbreviations

NVIC: the Netherlands society of intensive care
ZonMw: the Netherlands organization for health research and development
