# Supplementary figures and images for: Improvement of care for ICU patients with delirium by early screening and treatment: study protocol of iDECePTIvE study
Source: Implement Sci. 2014 Oct 2;9:143. doi: 10.1186/s13012-014-0143-7 (PMC4192432; doi:10.1186/s13012-014-0143-7)

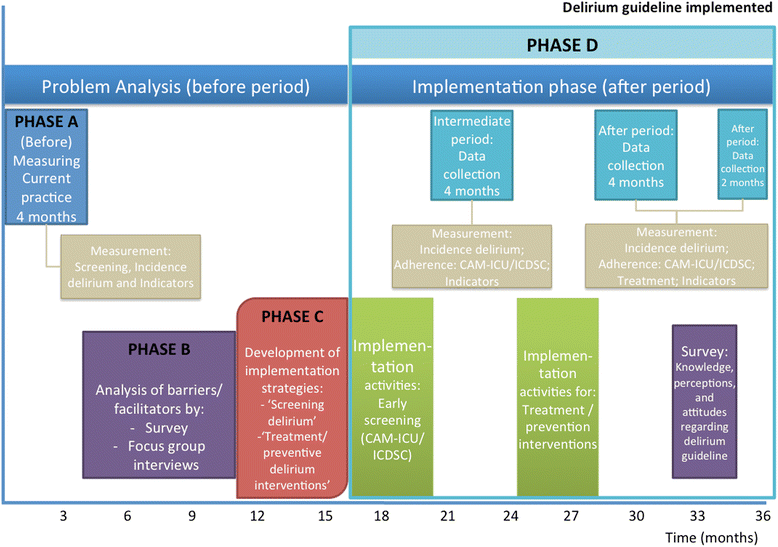

Supplement: Supplementary file 1 — Authors’ original file for figure 1 [file 13012_2014_143_MOESM1_ESM.gif]
